# Music and emotion

**DOI:** 10.1192/j.eurpsy.2021.2018

**Published:** 2021-08-13

**Authors:** I. Carneiro Silva, A. Gouveia, G. Dalagna, J.M. Oliveira, P. Carvalho, R. Costa, J. Gama

**Affiliations:** 1 Faculdade De Ciências Da Saúde, Universidade da Beira Interior / UBI, Covilhã, Portugal; 2 Departamento De Comunicação E Arte Da Universidade De Aveiro, Universidade de Aveiro, Aveiro, Portugal; 3 Faculdade De Ciências Sociais E Humanas, Universidade da Beira Interior, Covilhã, Portugal; 4 Cma - Centro De Matemática E Aplicações Da Ubi, Universidade da Beira Interior, Covilhã, Portugal

**Keywords:** Music, triggered, emotion, language

## Abstract

**Introduction:**

Music has been said to be emotion’s language. Research confirms a link between music structure and triggered emotions.

**Objectives:**

To assess the relationship between selected music excerpts and the emotions trigged, in order that the former will be used in future research.

**Methods:**

An anonymous study was performed in April 2019 on 65 subjects of both sexes, aged 19- 33 (mean=21,09; SD=3,05).Subjects listened 4 excerpts of music, believed to be related either to excitement or to calmness, and answered to a questionary on emotion’s triggered by each exposure.

**Results:**

Regarding to the music excerpts that were believed to induce excitement 80% of the subjects mentioned exciting emotions, 78% enjoyed the music while 78% didn’t knew them. For the ones that were believed to induce calmness 69% of the subjects mentioned calm emotions, 84% enjoyed the music and 62% didn’t knew the music. In an excerpt of music related to calmness, we observed association between knowing the music and the emotion trigged (p=0,027). The triggered emotion responses were independent of liking the music (P>0,05).

**Conclusions:**

In our study, independent of liking the music, the participants reported to have perceived the expected emotions triggered by musical excerpts, showing this to be a phenomenon related to music structure. Calmness perception may be also influenced by previous knowledge of the music and related experiences. The role of individual perceptions will be looked for in following studies.

**Disclosure:**

No significant relationships.

